# Burnout and resilience at work among health professionals serving in tertiary hospitals, in Ethiopia

**DOI:** 10.3389/fpubh.2023.1118450

**Published:** 2023-06-06

**Authors:** Yared Mulu Gelaw, Kashtan Hanoch, Bruria Adini

**Affiliations:** ^1^Department of Emergency and Disaster Management, Sackler Faculty of Medicine, School of Public Health, Tel-Aviv University, Tel Aviv, Israel; ^2^Department of Health Service Management, School of Public Health, College of Medicine and Health Sciences, Bahir Dar University, Bahir Dar, Ethiopia; ^3^Department of Surgery, Rabin Medical Center, Sackler Faculty of Medicine, Tel Aviv University, Tel Aviv, Israel

**Keywords:** health personnel, burnout, professional, resilience at work, Ethiopia

## Abstract

**Background:**

The quality of healthcare service is strongly affected by the health professionals’ levels of burnout and resilience at work (RaW). Developing resilience is a key component of medical professionalism. Although burnout and resilience are indicators used to assess the level of workplace hardship, there is a dearth of information in most developing countries, including Ethiopia.

**Objective:**

To assess the levels of burnout and ‘resilience at work’ among health professionals who work in the surgical care departments in teaching Ethiopian hospitals.

**Methods:**

A cross-sectional study design was applied among health professionals employed in surgical, gynecologic, and obstetric (Gyn/Obs) departments of two acute-care hospitals (n = 388). A structured self-administered English version questionnaire, consisting of validated scales to measure RaW and burnout, was used to collect the data;22 items of Maslach’s burnout inventory human service survey tool and 20 items of Win wood’s resilience at work’ measuring tool” was employed to assess the health professionals’ burnout level and Resilience at work, respectively. Linear logistics regression was employed for inferential statistical analysis to identify factors that predict RaW and burnout.

**Results:**

Burnout syndrome was shown among 101 (26.0%) study participants. Furthermore, 205 (52.8%), 150 (38.7%), and 125 (32.2%) participants presented high emotional exhaustion, high depersonalization, and low personal accomplishment, respectively. Emotional exhaustion was predicted by the participants’ profession, the hope of promotion, professional recognition, and workload. Depersonalization was predicted by age, profession, and perceived workload of the participants. The predictors for personal accomplishment were profession, relationship at work, professional recognition, and having a managerial position in addition to clinical duty. The participants’ mean RaW score was 78.36 (Standard deviation ±17.78). A negative association was found between RaW and emotional exhaustion and depersonalization. In contrast, a positive association was identified between RaW and personal accomplishment. The type of profession and marital status were positive predictors of RaW.

**Conclusion:**

A substantial amount of health professionals experience high burnout in one or more burnout dimensions. Level of RaW is more affected by burnout syndrome. Therefore, promoting activities that increase the level of professional RaW and recognition in their professional practice is needed to reduce job burnout. These findings are especially important concerning low socio-economic countries, as resilience is a vital component of the development of healthcare systems.

## 1. Introduction

Globally, the health workforce crisis is a common phenomenon that challenges the healthcare systems’ function (1, 2). Shortage of health workers, poor training modalities, maldistribution of human resources, and poor management of health professionals turnover constitutes a serious challenge for hospitals (1, 3). Workplace hardship in the health delivery set-up may result in professional burnout and poor ‘resilience at work’ (RaW), significantly challenging the health care system’s functioning (4). Clinical personnel who lack self-compassion and mindfulness frequently suffer from burnout and lower levels of RaW that impair the capacity to overcome difficulties (5).

Although there are no generally accepted definitions of burnout, the most commonly agreed definition is an extended exposure to chronic personal and interpersonal stressors on the job as characterized by three dimensions: exhaustion, depersonalization, and professional inefficacy (6). According to Christina Maslach et al., “emotional exhaustion” (EE) is described as the feeling of not being able to offer any more of oneself at an emotional level; “depersonalization” refers to a distant attitude toward work, the people being served by it and among colleagues; and “professional inefficacy” describes the feeling of not performing tasks adequately and of being incompetent at work (7).

Many medical professionals suffer from burnout, which is the psychological syndrome involving emotional exhaustion, feelings of helplessness, depersonalization, negative attitudes toward work and life, and reduced personal accomplishment (PA) (8). Burnout affects the quality-of-service delivery and exacerbates professional shortages in the health system by causing dropout from the place of work and/or profession (9–11).

Burnout is a protracted response to chronic and interpersonal stressors on the job (12), that has negative implications for job performance and social relationships (13). The level of burnout among medical professionals is nearly twice that of non-physician professionals (14). Burnout occurs when health professionals use ineffective coping strategies to protect themselves from work-related stress (15). Developing professional resilience may become an important strategy to minimize emotional distress, burnout, and work dropout. Many personal and demographic factors affect the medical personnel’s level of RaW.

According to Cooper et al., “resilience” is the ability of an individual to positively adjust to adversity, and can be applied to building personal strength (16). According to King et al. (17), resilience is the way individuals, groups/teams, and organizations respond to facing challenges and adversity. Southwick et al. (18) also defined resilience as “the capacity of a dynamic system to withstand or recover from significant disturbances.” It includes the steady trajectory of healthy functioning after an extremely hostile event.

In reliance on varied definitions of resilience, the term ‘resilience at work’, was also described as a dynamic capability that can allow individuals to thrive on challenges, given appropriate social and personal dimensions in their workplace (19). These dimensions include self-efficacy, self-control, the ability to engage in support and help, learning from difficulties, and persistence despite blocks to progress (20). Another definition of ‘resilience at work’ defined by Milton, was a “positive developmental trajectory characterized by demonstrated competence in the face of, and professional growth after, experiences of adversity in the workplace” (Page 3) (21). Resilience at work’ is an important attribute and one which can be learned and improved upon (22, 23).

Burnout is associated with health professionals facing unprofessional behavior, thoughts of suicide, retirement prematurely from their work, and errors during patient care (24). High stress and burnout reduce working performance and recovery from challenges, while highly resilient employees were found to be less affected by variations in working recovery (25, 26).

Ethiopia has been affected by a shortage of health professionals as well as high levels of professional burnout and low levels of resilience among healthcare workers. In response to the critical shortage of human resources in the healthcare system, the Government of Ethiopia invested significant resources in the effort to increase the quantity of the healthcare workforce, utilizing a ‘flood-and-retain strategy’. This strategy involves an accelerated and voluminous increase in the number of students studying health professions. In this regard, the number of health science colleges, as well as the enrollment of health professionals into higher institutions, has been increasing in the last 15 years (27). However, how many of those health professionals are resilient at work is to date not well known.

Therefore, this study is aimed to examine the level of burnout and ‘resilience at work’ among health professionals who are working at the obstetric/gynecologic and surgical care departments inTibebe-Ghion and the University of Gondar comprehensive Specialized referral Hospital (UoGSRH) in Ethiopia. The measurement will enable both the scientific realm and policymakers to understand the current level of burnout and RaW in the setting of a low socio-economic society and highlight components that should be managed to enhance the development of a more resilient healthcare system.

## 2. Materials and methods

### 2.1. Type of study and its courses

A facility-based, cross-sectional, quantitative study was conducted at two public teaching hospitals located in the Amhara region of Ethiopia (Tibebe-Ghion and Gondar teaching hospitals).

### 2.2. Investigated institutions

Bahir Dar University College of Medicine and Health Sciences was established in 2007 and is one of the youngest medical training institutions in Ethiopia authorized to provide medical and health professionals training in the past 10 years. The hospital that provides both teaching and clinical services under the college of medicine and health science is known as Tibebe-Ghion Campus. The hospital provides both outpatient and inpatient care services, with over 452 beds. A total of 621 health professionals, 128 intern general practitioners, and 211 residents are currently working in the hospital. The surgical and gynecological care department of the hospital consists of 167 clinical staff, of which 25 nurses and 28 surgeons work in the surgical care department. The rest (69 midwifery nurses and 19 gynecologists and obstetricians) work in the gynecologic department. Additionally, 26 different-level anesthesia providers currently work in the two departments.

The second study institution is the UoGSRH which is a teaching hospital under the Gondar University College of Medicine and Health Sciences; established in 1954. UoGSRH is one of the largest specialized hospitals in Ethiopia, with over 540 beds. A total of 986 clinical staff, 141 intern general practitioners, and 243 residents currently work in the hospital. The surgical and gynecological care departments of the hospital consist of 245 health professionals of which 37 nurses and 41 surgeons work in the surgical care department. The rest (101 midwifery nurses and 28 gynecologists and obstetricians) work in the gynecologic department. Additionally, 38 different-level anesthesia providers currently work in the two departments.

### 2.3. Study population

The study population included all physicians and residents from the surgical, gynecologic, and obstetric (Gyn/Obs) departments. Midwifery, anesthetic, and all other nursing specialties who are working in the operative sites of the aforementioned hospitals.

Health professionals who are planning to leave their institution due to completion of their residency program, intend to attend a training program in other hospitals, or due to any other reasons within the upcoming 2 years, were excluded from the study. Based on these eligibility criteria, a total of 412 clinical personnel (167 from Tibebe-Gion and 245 from UoGSRH) were eligible for the survey.

### 2.4. Study variables

Levels of professional burnout (low, medium, high) and levels of resilience at work (low, medium, high) were defined as the dependent variables. The type of profession, work experience, and demographic characteristics were collected as independent variables. Family size, income per family size, and behavioral factors such as chat chewing and cigarette smoking were examined as confounder variables.,

### 2.5. Study tool and reliability

To assess the health professionals’ burnout level, Maslach’s burnout inventory human service survey (MBI-HSS), a tool consisting of 22 items was used. The tool comprises emotional exhaustion (9 items), personal accomplishment (8 items), and depersonalization (5 items) with a seven-point response scale (0 to 6), ranging from 0 = never to 6 = daily (28). The total scores of each dimension were summed and categorized as low, moderate, or high, and the average score was also calculated. The cut-off point score for health personnel’s burnout was as follows: Emotional exhaustion: low (≤16), moderate (17–26), high (≥27); Personal accomplishment: low (≤33), moderate (29–34), high (>39); and Depersonalization: low (≤5), Moderate (6–9), High (≥10). Overall burnout (burnout syndrome) was considered when a health provider displayed high levels of emotional exhaustion and/or depersonalization and low levels of personal accomplishment (35, 36). In this study, the internal consistency of the MBI tool was checked. The overall internal consistency of the 22 items was high (Cronbach’s *α* = 0.87). Similarly, the domain-specific internal consistency was high for all three components, as follows: EE (Cronbach’s *α* = 0.89), Depersonalization (Cronbach’s *α* = 0.85), and PA domains (Cronbach’s *α* = 0.81).

Resilience at work was measured by using the Win wood ‘resilience at work’ measuring tool” (37). The tool consists of 20 items, classified into seven components with a seven-point response on a Likert scale (0 to 6), ranging from 0 = strongly disagree to 6 = strongly agree. The seven components are; Living authentically (three items), Finding one’s calling (four items), Maintaining perspective (three items), Managing stress (four items), Interacting cooperatively (two items), Staying healthy (two items), and Building networks (two items) (37). The total score of the scale was calculated to obtain a composite resilience value. The levels of resilience at work were calculated using the mean scores. Mean was used to determine whether the current score is lower, consistent, or higher than Win wood’s means core; participants who scored below 61, 61–81, and above 81 were considered as having a low, moderate, and high level of ‘resilience at work’ respectively (29, 38, 39). The internal consistency of the ‘resilience at work’ assessment tool was also assessed, which was high (Cronbach’s *α* = 0.89).

### 2.6. Study design

An English version of a self-administered structured questionnaire was used to collect the data on health professionals’ burnout and ‘resilience at work’. Before the actual data collection, the questionnaire was pre-tested among 20 health professionals (5% of the total sample). The pre-test study was conducted in other hospitals (not sampled in the study) that have similar characteristics to the main study participating hospitals.

Four data collectors and two supervisors participated in the data collection process. Intensive training was provided for the data collectors and supervisors before the data collection began. During the training, the trainers gave instructions concerning the questions to be asked, their meaning, ways to ask them, and how to record the answers. Both electronic and hard-copy survey tools were used to fill in the data. The hard copy was used for those participants who lack electronic access or were not interested in using it. The electronically filled data was uploaded directly to excel and exported to the statistical package for social sciences (SPSS, version 23) software for analysis. The hard-copy filled data were entered directly into SPSS-23.

The data collectors approached the respondents by self-introduction, explaining the objectives of the study as well as their autonomic participation in the study. After informed consent was received from each respondent, the questionnaire was distributed by one of the two data collection means. Furthermore, the data collectors supported respondents who needed further assistance during detailing and checked for any missing or incomplete information. For data collected using a hard copy, any missing or incomplete data were corrected by re-collecting the correct information before leaving the respondent.

During the data collection process, the supervisors traveled with the data collection teams, to observe and ensure that their teams provide self-introduction, and explain the objectives of the study, stressing the confidentiality of the information, and the anonymity of participating in the study. Moreover, the supervisors followed the data collectors to take informed consent from each respondent. They also checked and assisted if any additional training or clarifications were needed. Furthermore, the principal investigator checked all the data that was submitted from the field every other day and communicated as needed with the supervisors.

### 2.7. Data analysis

The collected data were checked for completeness and consistency. Consequently, the data was compiled, cleaned, coded, and then exported/entered into SPSS version 23 for analysis. A descriptive analysis was conducted to summarize the findings. Descriptive statistics comparison was done using a t-test and one-way analysis of variance (one-way ANOVA). Simple linear regression analysis was applied to select the candidate variables for the multiple linear regression model. To control the confounding effect, a variable with a value of *p* ≤0.2 on a simple linear regression was taken as a candidate variable for multiple linear regression. Multiple linear regression analysis was done *via* the enter method to identify the independent predictors for burnout for each dimension separately and for the RaW. value of *p* <0.05 on multiple linear regression analysis was declared a statistically significant predictor for each burnout dimension and resilience and unstandardized-β was used for interpretation. Multiple linear regression assumptions (normality, linearity, and constant variance) were checked. Linear-correlation analysis was used to test the correlation between the three dimensions of burnout and ‘resilience at work’. Additionally, t-test and one-way ANOVA were employed to test the differences in each burnout dimension and ‘resilience at work ‘according to the participants’ demographic and work-related characteristics.

## 3. Results

### 3.1. Participants’ socio-demographic characteristics

A total of 388 health professionals participated in the survey with a response rate of 94.2%. Around three-fourths of the respondents, 287 (74.0%) were male. The median age with Interquartile Range (±IQR) of the respondents was 29 (±5) years of age, which ranged from 20 to 49 years. Almost half of the participants, 197 (50.8%) were married. Three hundred thirty-eight (87.1%) respondents were Orthodox Christian followers. Professionally, more than one-third, 142 (36.6%) of the respondents were residents, and 264 (68.0%) respondents have 5 years or below of work experience. The average salary of the respondents in Ethiopian Birr (EBR) was 10,653.6 with a standard deviation (SD) of ±4532.7, while 194 (50.0%) respondents had a monthly salary of 10,075EBR or below (Table 1).

**Table 1:** Socio-demographic characteristics of health personnel working at the surgical care department; tertiary hospitals, North-West Ethiopia, 2021 (*n* = 388).

Variables	Category	Total *N* (%)	Tibebe Ghion *n* (%)	Gondar *n* (%)	Value of *p*
Sex	Male	287 (74.0)	128 (85.3)	159 (66.8)	0.000
	Female	101 (26.0)	22 (14.7)	79 (33.2)	
Age	20–25	41 (10.6)	13 (8.7)	28 (11.8)	0.732
	26–30	215 (55.4)	85 (56.6)	130 (54.6)	
	31–35	90 (23.2)	37 (24.7)	53 (22.3)	
	>35	42 (10.8)	15 (10.0)	27 (11.3)	
Marital status	Single	185 (47.7)	68 (45.3)	117 (49.2)	0.091
	Married	197 (50.8)	82 (54.7)	115 (48.3)	
	Others (divorced and widowed)	6 (1.5)	0	6 (2.5)	
Religion	Orthodox Christian	338 (87.1)	130 (86.7)	208 (87.4)	0.029
	Muslim	33 (8.5)	18 (12.0)	15 (6.3)	
	Protestant	13 (3.4)	2 (1.3)	11 (4.6)	
	Others	4 (1.0)	0	4 (1.7)	
Profession	Specialist	76 (19.6)	31 (20.7)	45 (18.9)	0.000
	Resident	142 (36.6)	56 (37.3)	86 (36.1)	
	Midwifery	37 (9.5)	0	37 (15.5)	
	Nurse	83 (21.4)	41 (27.3)	42 (17.6)	
	Anesthetist	50 (12.9)	22 (14.7)	27 (11.3)	
Work experience	≤2 years	96 (24.7)	43 (28.7)	53 (22.3)	0.360
	3–5 years	168 (43.3)	61 (40.7)	107 (44.9)	
	>5 years	124 (32.0)	46 (30.6)	78 (32.8)	
Have a managerial position	Yes	67 (17.3)	25 (16.7)	42 (17.6)	0.891
	No	321(82.7)	125 (83.3)	196 (82.4)	
Monthly salary	≤7,071	107 (27.6)	45 (30.0)	62 (26.1)	0.000
	7,072–10,075	87 (22.4)	12 (8.0)	75 (31.5)	
	10,076-11,305	101 (26.0)	57 (38.0)	44 (18.5)	
	≥11,306	93 (24.0)	36 (24.0)	57 (23.9)	
Have children under 18 years old	Yes	112 (28.9)	52 (34.7)	60 (25.2)	0.051
	No	276 (71.1)	98 (65.3)	178 (74.8)	
Number of children<18 years old (*n* = 112)	One	54 (48.2)	22 (43.1)	32 (52.5)	0.348
	Two and more	55 (51.8)	29 (56.9)	29 (47.5)	

### 3.2. Working environment-related characteristics

A majority (*N* = 379, 97.7%) of the respondents reported that they have a good or neutral relationship at work. Three-hundred thirty-one (85.3%) respondents perceived that there is a high workload, while 294 (75.8%) respondents had good or neutral perceptions of the existing management system in their hospitals. Regarding the perception of the working environment, 250 (64.4%) of the respondents have neutral or unsuitable perceptions. More than two-thirds, (*N* = 257, 66.2%) of the respondents reported that they fear contracting an illness during work (Table 2).

**Table 2:** Working environment-related characteristics of health personnel working at the surgical care department; tertiary hospitals, North-West Ethiopia, 2021 (*n* = 388).

Variables	Category	Total *N* (%)	Tibebe Ghion *n* (%)	Gondar *n* (%)	*p*-value
Relationship at workplace	Good	350 (90.2)	132 (88.0)	218 (91.6)	0.499
	Neutral	29 (7.5)	14 (9.3)	15 (6.3)	
	Low	9 (2.3)	4 (2.7)	5 (2.1)	
Perception of the management system	Good	207 (53.4)	49 (32.7)	158 (66.4)	0.000
	Neutral	87 (22.4)	38 (25.3)	49 (20.6)	
	Low	94 (24.2)	63 (42.0)	31 (13.0)	
Prospect of promotion	Good	258 (66.5)	73 (48.7)	185 (77.7)	0.000
	Neutral	87 (22.4)	48 (32.0)	39 (16.4)	
	Low	43 (11.1)	29 (19.3)	14 (5.9)	
Perception of workload	High	331 (85.3%)	127 (84.7)	204 (85.7)	0.047
	Balanced	46 (11.9)	15 (10.0)	31 (13.0)	
	Low	11 (2.8)	8 (5.3)	3 (1.3)	
Perception of the working environment	Suitable	138 (35.6)	23 (15.3)	115 (48.3)	0.000
	Neutral	90 (23.2)	30 (20.0)	60 (25.2)	
	Unsuitable	160 (41.2)	97 (64.7)	63 (26.5)	
Perception of professional recognition	Good	236 (60.8)	73 (48.7)	163 (68.5)	0.000
	Neutral	68 (17.5)	27 (18.0)	41 (17.2)	
	Low	84 (21.7)	50 (33.3)	34 (14.3)	
Resource availability	Sufficient	80 (20.6)	12 (8.0)	68 (28.6)	0.000
	Neutral	41 (10.6)	14 (9.3)	27 (11.3)	
	Insufficient	267 (68.8)	124 (82.7)	143 (60.1)	
Is there any fear of contracting an illness during work	Yes	257 (66.2)	127 (84.7)	130 (54.6)	0.000
	No	131 (33.8)	23 (15.3)	108 (45.4)	

### 3.3. Magnitude of burnout

**Figure 1:**
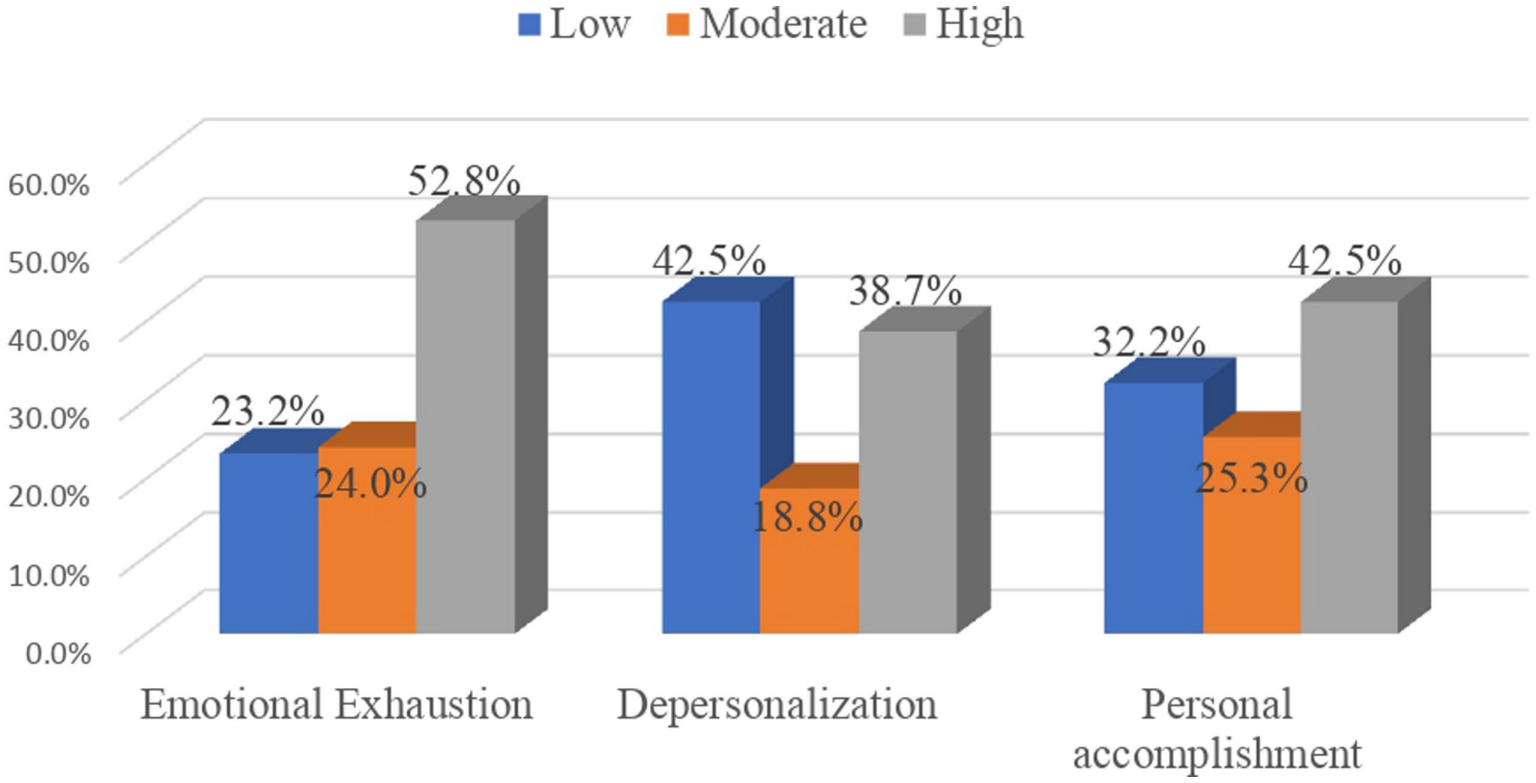
Maslach burnout subscale levels among health personnel working at the surgical care department; tertiary hospitals, north-west Ethiopia, 2021 (*n* = 388).

Burnout syndrome was shown among 101 (26.0%) study participants; which means they displayed high burnout in emotional exhaustion and/or depersonalization and low personal accomplishment, and 318 (82.0%) of them experienced burnout for at least one dimension. Furthermore, 205 (52.8%), 150 (38.7%), and 125 (32.2%) participants presented high emotional exhaustion, high depersonalization, and low personal accomplishment respectively, i.e., displayed a high level of burnout (Figure 1). The participants’ mean score with standard-deviation (±SD) of emotional exhaustion, depersonalization, the personal accomplishment was 27.28 ± 12.67, 9.78 ± 7.93, and 36.19 ± 7.80, respectively.

### 3.4. Comparison of burnout sub-scales according to participants’ demographic characteristics

Table 3 shows the comparison of the participants’ burnout levels according to the demographic and work-related variables.

**Table 3:** The mean score of the three burnout dimensions based on the participants demographic and work-related variables of health personnel working at the surgical care department; tertiary hospitals, North-West Ethiopia; 2021 (*n* = 388).

Variable	Burnout dimensions					
	Emotional exhaustion		Depersonalization		Personal accomplishment	
	Mean (±SD)	*P*-value	Mean (±SD)	*P*-value	Mean (±SD)	*P*-value
Gender						
Male	28.00 (12.29)	0.059	9.26 (7.91)	**0.030**	36.74 (7.39)	**0.019**
Female	25.23 (15.86)		11.25 (7.83)		34.62 (8.71)	
Age						
≤ 25	27.95 (14.22)	0.353	10.24 (8.52)	0.122	35.71 (7.45)	0.704
25–30	28.14 (12.08)		9.17 (7.50)		35.96 (8.05)	
31–35	25.80 (12.46)		9.82 (7.44)		37.03 (7.18)	
> 35	25.38 (14.42)		12.33 (10.01)		36.02 (8.22)	
Religion						
Christian	27.46 (12.50)	0.350	10.07 (8.01)	**0.017**	35.88 (7.96)	**0.011**
Muslim	25.30 (14.49)		6.64 (6.25)		39.48 (4.67)	
Marital status						
Single	28.54 (12.47)	0.120	8.88 (7.50)	**0.024**	36.38 (7.88)	0.598
Married	25.99 (12.62)		10.41 (8.09)		36.11 (7.70)	
Others	30.50 (18.58)		16.17 (11.50)		33.17 (9.43)	
Profession						
Specialist	24.33 (13.65)	0.133	8.63 (7.58)	**< 0.001**	36.25 (7.51)	**<0.001**
Residents	28.42 (11.77)		7.76 (6.48)		38.03 (6.54)	
Midwifery	26.73 (14.22)		15.14 (9.36)		32.54 (8.55)	
Nurse	29.58 (13.05)		8.88 (8.17)		36.76 (7.01)	
Anesthetist	26.89 (12.08)		12.42 (8.06)		34.28 (9.20)	
Hospital in which they are employed						
Bebhionn	27.33 (12.11)	0.953	8.49 (6.95)	0.067	36.26 (7.33)	0.890
Gondar	27.25 (13.05)		10.58 (8.40)		36.15 (8.10)	
Work experience						
≤2 years	28.93 (11.01)	0.216	8.86 (7.24)	0.220	36.3 (7.65)	0.983
3–5 years	27.35 (13.13)		9.61 (7.81)		36.19 (7.64)	
≥ 6 years	25.91 (13.20)		10.70 (8.55)		36.10 (8.18)	
Have managerial position						
Yes	26.00 (12.39)	0.365	10.51 (7.99)	0.407	33.16 (8.60)	**<0.001**
No	27.55 (12.74)		9.62 (7.92)		36.82 (7.48)	
Have <18 years of children						
Yes	24.31 (13.08)	**0.003**	9.31 (7.33)	0.464	36.04 (7.89)	0.815
No	28.48 (12.34)		9.96 (8.17)		36.25 (7.78)	
Monthly income in EBR						
≤ 7,071	25.87 (12.71)	**0.001**	12.01 (8.18)	**< 0.001**	32.28 (8.74)	**<0.001**
7,072–10,075	31.34 (10.51)		10.72 (8.43)		37.11 (6.88)	
10,076–11,305	28.11 (12.73)		7.76 (6.73)		38.21 (7.09)	
≥ 11,306	24.19 (13.51)		8.51 (7.68)		36.48 (7.33)	
Relationship at work						
Good	26.74 (12.84)	**0.024**	9.95 (8.01)	0.383	36.32 (7.90)	0.240
Neutral	31.10 (9.87)		7.83 (6.93)		35.97 (6.91)	
Poor	35.89 (9.96)		9.44 (7.92)		31.89 (5.53)	
Management system						
Good	26.01 (12.95)	**0.033**	10.93 (8.16)	**0.004**	36.12 (8.25)	0.698
Neutral	27.22 (12.68)		7.74 (7.00)		36.77 (7.10)	
Poor	30.13 (11.71)		9.12 (7.85)		35.81 (7.43)	
Hope of promotion						
Good	25.64 (12.68)	**0.001**	10.26 (8.10)	0.237	36.49 (7.98)	**0.020**
Neutral	29.66 (12.17)		8.75 (7.72)		36.84 (6.86)	
Poor	32.30 (11.81)		8.95 (7.20)		33.09 (7.93)	
Perception of workload						
High	28.39 (12.45)	**<0.001**	9.82 (8.00)	0.375	36.40 (7.61)	0.424
Balanced	20.70 (11.21)		10.20 (7.87)		35.15 (8.72)	
Low	21.27 (16.07)		6.55 (5.52)		34.27 (9.35)	
Perception of the working environment						
Suitable	24.63 (13.10)	**0.008**	11.07 (8.04)	0.055	35.44 (8.54)	0.151
Neutral	28.12 (10.62)		8.83 (7.62)		37.49 (7.03)	
Not suitable	29.09 (13.05)		9.19 (7.91)		36.11 (7.80)	
Professional recognition						
Good	25.61 (12.56)	**0.004**	10.75 (8.12)	**0.007**	36.77 (7.91)	**0.021**
Neutral	29.32 (12.90)		8.96 (8.45)		36.75 (7.46)	
Poor	30.32 (12.16)		7.71 (6.47)		34.11 (7.47)	
Resource availability						
Sufficient	23.63 (13.42)	**0.009**	12.08 (8.63)	**0.014**	35.79 (8.84)	0.274
Neutral	30.12 (11.71)		8.98 (8.15)		38.02 (6.35)	
Insufficient	27.94 (12.41)		9.21 (7.57)		36.03 (7.66)	
Have a fear of contracting an illness during work						
Yes	29.33 (12.22)	**<0.001**	9.11 (7.49)	**0.021**	36.26 (7.54)	0.815
No	23.24 (12.65)		11.08 (8.61)		36.06 (8.31)	

The dependent variable has a difference according to the independent variable that has such a bold *p*-value.

Using the independent *t*-test statistical analysis, a higher mean score of emotional exhaustion was found among participants who did not have children under 18 years compared to those who had such young children (28.48 ± 12.34 vs. 24.31 ± 13.08 respectively; *p* < 0.01). The analysis of variance (ANOVA) showed a significant difference in the mean score of EE by participants’ monthly income; most especially, the difference was noted between respondents whose monthly income was above 11,306 EBR or between 7,072–10,075 EBR (24.19 ± 13.51 vs. 31.34 ± 10.51 respectively; *p* < 0.01). Participants who had a poor relationship at work had a higher mean score of EE compared to those who had a good relationship at work (35.89 ± 9.96 vs. 26.74 ± 12.84 respectively; *p* = 0.024). A high mean score of EE was also observed among participants who had a high perceived workload compared to those who had a low perceived workload (28.39 ± 12.45 vs. 21.27 ± 16.07 respectively; *p* < 0.01). Moreover, participants who had poor hope of promotion had a high EE mean score compared to those who had good hope of promotion (32.30 ± 11.81 vs. 25.64 ± 12.68 respectively; *p* < 0.01). Relatively, a higher EE mean score was found among males compared to females (28.00 ± 12.29 vs. 25.23 ± 15.86 respectively) but this difference was not found to be significant (*p* > 0.05). The mean score of EE did not show a significant difference by the type of hospital in which the clinicians were employed (27.33 ± 12.11 for Tibebe-Ghion vs. 27.25 ± 13.05 for Gondar; *p* > 0.05).

Burnout levels in the dimension of depersonalization were higher among females compared to males (11.25 ± 7.83 vs. 9.26 ± 7.91 respectively; *p* = 0.030). The mean score of depersonalizations was also higher among Christians compared to Muslims (10.07 ± 8.01 vs. 6.64 ± 6.25 respectively; *p* = 0.017). There was a significant difference in the mean score of depersonalizations by profession; most especially the difference was observed between midwifery and residents (15.14 ± 9.36 vs. 7.76 ± 6.48 respectively; *p* < 0.01). A significant difference in the mean score of depersonalizations was also noted concerning the participants’ monthly income; particularly, these differences were noted between participants who received a monthly income below 7,071 EBR compared to those who earn 10,076–11305EBR (12.01 ± 8.16 vs. 7.76 ± 6.73 respectively; *p* < 0.01). However, the difference in the mean score of depersonalizations was not observed by the participants’ work experience, having children under 18 years old, relationships at work, and hope of promotion (*p* > 0.05).

Concerning burnout levels in the dimension of personal accomplishment, a higher mean score was reported among males compared to females (36.74 ± 7.39 vs. 34.62 ± 8.71 respectively; *p* = 0.019), and among Muslim religion, followers compared to Christians (39.48 ± 4.68 vs. 35.88 ± 7.96 respectively; *p* = 0.011). Significant differences in the mean score of PA were found according to the profession of the respondents; most especially the difference was noted between residents and midwifery as well as between specialists and midwifery (38.03 ± 6.54 for residents, 36.25 ± 7.51 for specialists’ vs. 32.54 ± 8.55 for midwifery; *p* < 0.01). The mean score of PA was lower among respondents who hold a managerial position compared to those who do not hold such positions (33.16 ± 8.60 vs. 36.82 ± 7.48 respectively; *p* < 0.01). Significant differences in PA mean score were also observed in participants ‘monthly income; most notably, it was lower among respondents whose monthly income was below 7,071 EBR compared to those whose monthly income was between 10,076–11,305 EBR (32.28 ± 8.74 vs. 38.21 ± 7.09 respectively; *p* < 0.01). From the work-related variables, there was a significant difference in the mean score of PA according to participants’ perceived hopes of promotion and professional recognition. Particularly, participants who have poor hope of promotion have lower levels compared to those who have good and neutral levels of hope (33.09 ± 7.93 vs. 36.49 ± 7.98 and 36.84 ± 6.86 respectively; *p* = 0.020). The same trend was identified among those who have a poor perception of professional recognition compared to those with a good perception (34.11 ± 7.47 vs. 36.77 ± 7.91 respectively; *p* = 0.021).

The three burnout domains were not found to be significantly different according to the participant’s age, work experience, or the hospital in which they are employed (*p* > 0.05; Table 3).

### 3.5. The magnitude of ‘resilience at work’ (RaW)

The participants’ mean resilience at work score with standard deviation (±SD) was 78.36 ± 17.78. In this study, 53 (13.7%), 141 (36.3%), and 194 (50.0%) respondents have low, moderate, and high levels of resilience at work, respectively.

The mean resilience score has a significant difference across the participants’ marital status, profession, and monthly income. Married women have a high resilience mean score compared to those who are not married (either divorced or widowed together) (79.18 ± 18.20 vs. 60.50 ± 26.58 respectively; *p* = 0.038). Professionally, specialists have the highest (82.59 ± 14.17) resilience mean scores compared to other professionals, and the lowest level of resilience at work was found among Midwives (70.62 ± 21.04; *p* < 0.001). Similarly, the mean score of resilience was found to be higher according to the income level of the respondents. The mean score for income <7,071, 7,072–10,075, 10,076–11,305, and > 11,305 was 74.04 ± 21.45, 76.94 ± 16.17, 80.66 ± 16.21, and 82.17 ± 15.03 respectively; *p* < 0.01.

Resilience at work had no significant difference according to the hospital in which the clinicians are employed (77.64 ± 16.00 for Tibebe-Ghion vs. 78.82 ± 18.84 for Gondar hospital; *p* > 0.05). Relatively, the mean score of resilience at work was higher among participants in the age group between 31 and 35 years compared to those whose age was below 26 years (82.59 ± 15.22 vs. 76.05 ± 18.18 respectively; *p* > 0.05). Resilience at work had no significant difference by the sex of the respondents (77.34 ± 20.57 for females vs. 78.82 ± 16.72 for males; *p* > 0.05). Moreover, the score of resilience at work had no significant difference according to the respondents’ work experience, managerial position, workload, working environment’s suitability for work, and resource availability (*p* > 0.05; Table 4).

**Table 4:** Mean scores of RaW according to the participants’ demographic and working environmental-related variables of health personnel working at the surgical care department; tertiary hospitals, North-West Ethiopia; 2021 (*n* = 388).

Variables	Category	Mean (±SD)	*p*-value
Age	≤ 25 years	76.05 (18.18)	0.072
	26–30	77.04 (18.46)	
	31–35	82.59 (15.22)	
	≥ 36	78.33 (18.07)	
Gender	Female	77.34 (20.57)	0.501
	Male	78.72 (16.72)	
Hospital they are serving	Tibebe-Ghion	77.64 (16.00)	0.525
	Gondar	78.82 (18.84)	
Have a managerial position	No	78.74 (17.45)	0.360
	Yes	76.55 (19.35)	
Marital status	Single	78.07 (16.87)	**0.038**
	Married	79.18 (18.20)	
	Others (divorced and widowed)	60.50 (26.58)	
Religion	Christian	78.03 (18.01)	0.232
	Muslim	81.91 (14.93)	
Profession	Specialist	82.59 (14.17)	**0.007**
	Resident	79.77 (15.62)	
	Midwifery	70.62 (21.04)	
	Anesthetist	78.06 (15.71)	
	Nurse	75.71 (22.21)	
Monthly income in Ethiopian Birr	≤ 7,071	74.04 (21.45)	**0.005**
	7,072–10,075	76.94 (16.17)	
	10,076–11,305	80.66 (16.21)	
	≥ 11,306	82.17 (15.03)	
Work experience	≤ 2 years	77.26 (18.16)	0.660
	3–5 years	78.20 (17.40)	
	≥ 6 years	79.44 (18.08)	
Have <18 years of children	No	77.68 (17.65)	0.236
	Yes	80.04 (18.07)	
Perceived relationship at work	Good	78.68 (17.89)	0.541
	Neutral	76.03 (15.96)	
	Poor	73.67 (19.63)	
Perception of the management system	Good	78.86 (19.24)	0.450
	Neutral	79.34(16.89)	
	Poor	76.37 (15.05)	
Hope of promotion	Good	79.40 (18.64)	0.078
	Neutral	78.05 (14.59)	
	Poor	72.81 (17.68)	
Perception of workload	High	77.90 (17.67)	0.202
	Balanced	82.54 (18.78)	
	Low	74.82 (15.53)	
Perception of the working environment	Suitable	78.68 (20.77)	0.176
	Neutral	80.96 (14.22)	
	Not suitable	76.63 (16.67)	
Perception of professional recognition	Good	80.22 (18.50)	**0.036**
	Neutral	75.12 (16.38)	
	Poor	75.76 (16.22)	
Perception of resource availability	Sufficient	77.60 (21.97)	0.627
	Neutral	80.80 (18.60)	
	Insufficient	78.22 (16.24)	
Have a fear of contracting an illness during work	No	80.47 (18.28)	0.096
	Yes	77.29 (17.46)	

The dependent variable has a difference according to the independent variable that has such a bold *p*-value.

### 3.6. Correlation between burnout sub-scales and resilience at work

Resilience at work was found to be associated with all three burnout dimensions (Pearson correlation between −0.139 to 0.479; *p* < 0.05). The Pearson correlation analysis showed that resilience at work has a negative association with emotional exhaustion and depersonalization, and in contrast, a positive association with the personal accomplishment burnout sub-scale.

### 3.7. Factors associated with burnout and ‘resilience at work’

#### 3.7.1. Factors associated with emotional exhaustion

**Figure 2:**
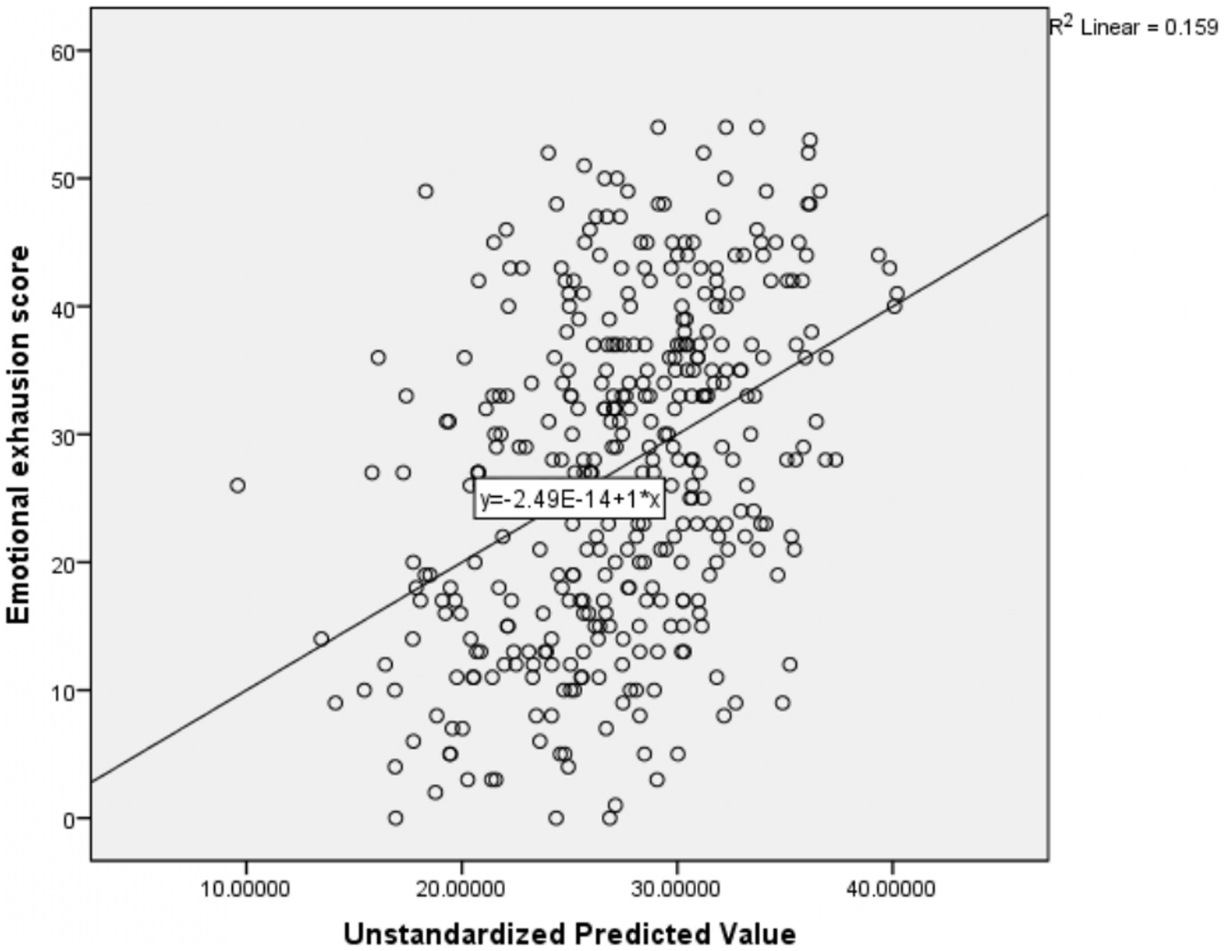
Scatter plot showing the amount of mean variation of emotional exhaustion score explained by the independent variables together.

Multiple linear regression results revealed that midwifery professionals [*β*: 5.503, 95%CI: 0.125, 10.882], anesthetic professionals [*β*: 5.029, 95%CI: 0.260, 9.798], the hope of promotion [*β*: 1.688, 95%CI: 0.039, 3.336], perception of professional recognition [*β*: 1.568, 95%CI: 0.120, 3.105] and fear of contracting illness during work [*β*: 4.426, 95%CI: 1.662, 7.190] were positively associated with the emotional exhaustion score. In contrast, the perception of workload [*β*: -3.367, 95%CI: −4.997, −1.736] was negatively associated with the emotional exhaustion score (Table 5). The R-square of this regression model was 0.159; which means that 15.9% of the dependent variable (emotional exhaustion) mean variation is explained by these independent variables collectively (Figure 2).

**Table 5:** Factors associated with emotional exhaustion among health personnel working at the surgical care department; tertiary hospitals, North-West Ethiopia; 2021 (*n* = 388).

Variables	Unstandardized β-coefficient	95% CI		*p*-value
		Lower	Upper	
Age (20–49 years)	0.362	−0.133	0.857	0.151
Work experience (< 1 to 31 years)	−0.449	−0.976	0.079	0.095
Gender (male)	0.284	−2.928	3.496	0.862
Marital status				
Married	1	1	11	
Single	1.351	−1.412	4.114	0.337
Others (divorced and widowed)	4.490	−5.318	14.299	0.369
Profession				
Specialist	1	1	1	1
Resident	2.642	−1.298	6.581	0.188
Midwifery	5.503	0.125	10.882	**0.045**
Anesthetist	5.029	0.269	9.798	**0.039**
Nurse	3.145	−1.223	7.513	0.158
Relationship at the workplace ^a^ (1-5)	1.062	−0.651	2.775	0.224
Perception of management system ^a^ (1-5)	−0.578	−1.957	0.802	0.411
The hope of promotion ^a^ (1-5)	1.688	0.039	3.336	**0.045**
Perception of workload ^b^ (1-5)	−3.367	−4.997	−1.736	**< 0.001**
Perception of working Environment ^c^ (1-5)	−0.295	−1.816	1.225	0.703
Perception of professional recognition ^a^ (1-5)	1.568	0.120	3.015	**0.034**
Resource availability ^d^ (1-5)	0.597	−0.784	1.979	0.396
Fear of contracting an illness during work (yes)	4.426	1.662	7.190	**0.002**

^a^(Very good, good, neutral, bad and very bad).

^b^(Very high, high, balanced, low and very low).

^c^(Very suitable, suitable, neutral, unsuitable and very unsuitable).

^d^(highly sufficient, sufficient, neutral, insufficient, highly insufficient).

*R* = 0.394, *R*^2^ = 0.159, *p* < 0.001.

The dependent variable has a difference according to the independent variable that has such a bold *p*-value.

#### 3.7.2. Factors associated with depersonalization

**Figure 3:**
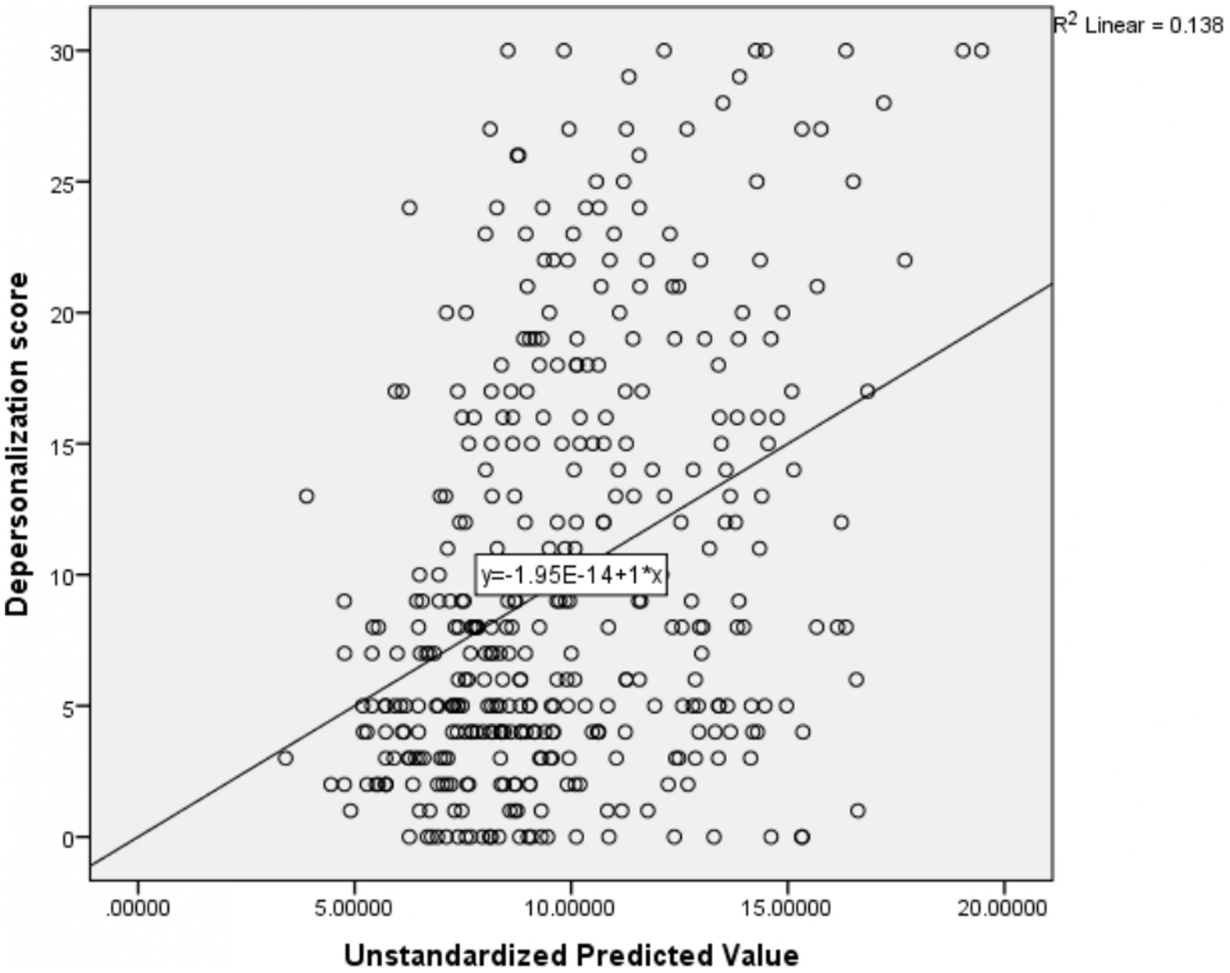
Scatter plot showing the amount of mean variation of Depersonalization score explained by the independent variables together.

The multiple linear regression results revealed that depersonalization was positively affected by the participants’ age [*β*: 0.235, 95% CI:0.016, 0.455] and professional type; midwifery [*β*:7.032, 95% CI: 3.627, 10.437], and nursing profession [*β*:4.754, 95% CI: 2.075,7.433]. Conversely, it was negatively affected by the participants’ perception of workload [*β*: -1.184, 95% CI: −2.207, −0.161] (Table 6). The R-square of this regression model was 0.138; these independent variables collectively explained 13.8% of the dependent variable (depersonalization) mean variation (Figure 3).

**Table 6:** Factors associated with depersonalization among health personnel working at the surgical care department; tertiary hospitals, North-West Ethiopia; 2021 (*n* = 388).

Variables	Unstandardized β-coefficient	95% CI		*p*-value
		Lower	Upper	
Age	0.235	0.016	0.455	**0.036**
Gender (male)	0.424	−1.591	2.438	0.679
Profession				
Specialist	1	1	1	
Resident	−0.175	−2.566	2.215	0.886
Midwifery	7.032	3.627	10.437	**< 0.001**
Anesthetist	1.281	−1.587	4.150	0.380
Nurse	4.754	2.075	7.433	**0.001**
The hope of promotion^a^ (1-5)	−0.139	−0.999	0.772	0.752
Perception of workload^b^ (1-5)	−1.184	−2.207	−0.161	**0.023**
Resource availability^d^ (1-5)	−0.317	−1.131	0.497	0.445
Have fear of contracting an illness during work (yes)	−0.709	−2.456	1.038	0.425
The hospital they are serving				
Gondar	0.982	−0.783	2.746	0.275
Tibebe Ghion	1	1	1	

*R* = 0.371, *R*^2^ = 0.138, *p* < 0.001.a(Very good, good, neutral, bad and very bad). b(Very high, high, balanced, low and very low). d(highly sufficient, sufficient, neutral, insufficient, highly insufficient).The dependent variable has a difference according to the independent variable that has such a bold *p*-value.

#### 3.7.3. Factors associated with personal accomplishment

**Figure 4:**
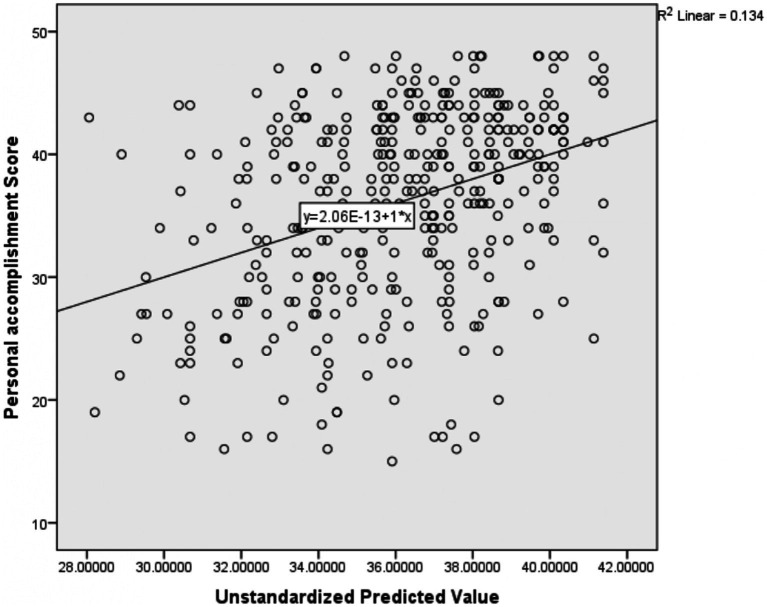
Scatter plot showing the amount of mean variation of personal accomplishment score explained by the independent variables together.

The results of the multiple linear regression indicated that midwifery professionals [: -4.103, 95%CI: −7.105, −1.100], have managerial positions [: -3.806, 95%CI: −5.783, −1.819], relationship at workplace (very good, very bad) [*β*: -1.431, 95%CI: −2.447, −0.414], and perception of professional recognition (very good, very bad) [*β*: -1.038, 95%CI: −1.856, −0.221] were negatively associated with the personal accomplishment score (Table 7). The *R*-square of this regression model was 0.134; these independent variables collectively explained 13.4% of the mean variation of personal accomplishment (Figure 4).

**Table 7:** Factors associated with personal accomplishment among health personnel working at the surgical care department; tertiary hospitals, North-West Ethiopia; 2021 (*n* = 388).

Variables	Unstandardized β-coefficient	95% CI		*p*-value
		Lower	Upper	
Gender (male)	1.282	−0.604	3.169	0.182
Profession				
Specialist	1	1	1	
Resident	0.626	−1.487	2.739	0.560
Midwifery	−4.103	−7.105	−1.100	**0.008**
Anesthetist	0.216	−2.446	2.879	0.873
Nurse	−2.280	−4.695	0.135	0.064
Have a managerial position (yes)	−3.806	−5.783	−1.819	**< 0.001**
Relationship at the workplace^a^ (1-5)	−1.431	−2.447	−0.414	**0.006**
The hope of promotion^a^ (1-5)	−0.248	−1.141	0.645	0.586
Perception of professional recognition^a^ (1-5)	−1.038	−1.856	−0.221	**0.013**

*R* = 0.342, *R*^2^ = 0.134, *p* < 0.001.a(Very good, good, neutral, bad, and very bad).The dependent variable has a difference according to the independent variable that has such a bold *p*-value.

#### 3.7.4. Factors associated with ‘resilience at work’

**Figure 5:**
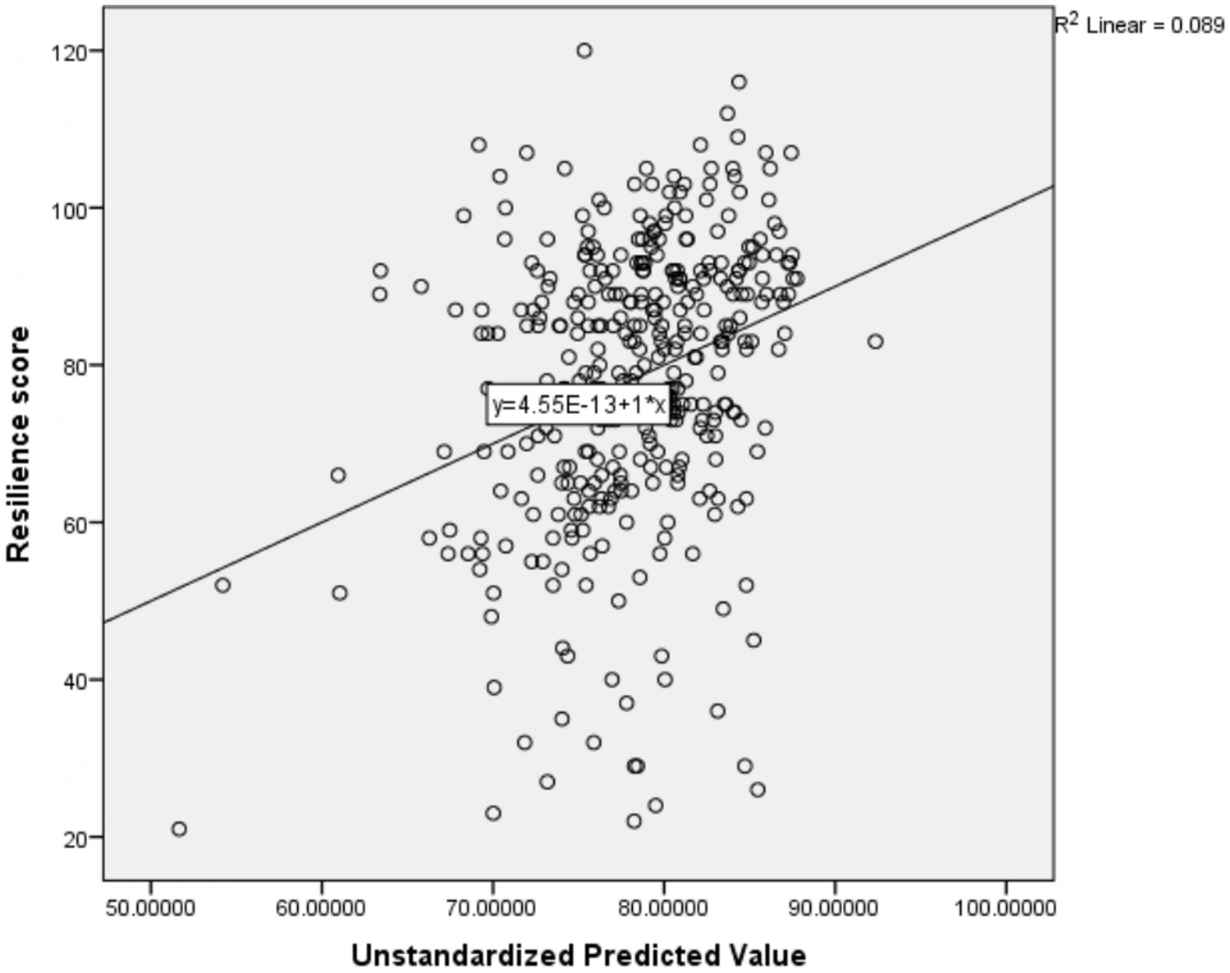
Scatter plot showing the amount of mean variation of resilience at work score explained by the independent variables together.

The multiple linear regression results indicated that the level of ‘resilience at work’ was affected by the participants’ profession and marital status. Participants with midwifery [: -12.258, 95%CI: −19.888, −4.627], BSc nurse [*β*: -6.657, 95%CI: −12.616, −0.699], and others (divorced and widowed) marital status category [*β*: -16.410, 95%CI: −30.578, −2.243] were negatively associated with the participants ‘resilience at work’ score (Table 8). The R-square of this regression model was 0.089; these independent variables explained 8.9% of resilience at work mean variation score (Figure 5).

**Table 8:** Factors associated with ‘resilience at work’ among health personnel working at the surgical care department; tertiary hospitals, north-west Ethiopia; 2021 (*n* = 388).

Variables	Unstandardized β-coefficient	95% CI		*p*-value
		Lower	Upper	
Age	0.140	−0.361	0.641	0.583
Profession				
Specialist	1	1	1	
Resident	−2.816	−8.368	2.735	0.319
Midwifery	−12.258	−19.888	−4.627	**0.002**
Anesthetist	−3.065	−9.759	3.629	0.369
Nurse	−6.657	−12.616	−0.699	**0.029**
Marital status				
Married	1	1	1	
Single	−0.797	−4.755	3.161	0.692
Others (divorced and widowed)	−16.410	−30.578	−2.243	**0.023**
Relationship at the workplace^a^ (1-5)	−1.317	−3.771	1.138	0.292
The hope of promotion^a^ (1-5)	−1.173	−3.304	0.959	0.280
Perception of working Environment^c^ (1-5)	0.022	−1.958	2.001	0.983
Perception of professional recognition^a^ (1-5)	−1.753	−3.819	0.314	0.096
Have fear of contracting an illness during work (yes)	−2.594	−6.505	1.317	0.193

*R* = 0.299, *R*^2^ = 0.089, *p* < 0.001.a(Very good, good, neutral, bad, and very bad). c(Very suitable, suitable, neutral, unsuitable and very unsuitable).The dependent variable has a difference according to the independent variable that has such a bold *p*-value.

## 4. Discussion

In this study, 23.2, 24.0, and 52.8% of study participants have a low, moderate, and high level of burnout in Emotional exhaustion, respectively. Regarding burnout in depersonalization, 42.5, 18.8, and 38.7% of participants have low, moderate, and high levels of burnout, respectively. Similarly, 32.2, 23.5%, and 42. % of participants have low, moderate, and high levels of burnout in personal accomplishment. Regarding resilience at work, 13.7, 36.3, and 50.0% of respondents have low, moderate, and high levels of resilience at work, respectively. Resilience at work has a positive association with PA and an inverse association with EE and depersonalization burnout sub-domains.

### 4.1. Level of burnout

The study revealed that more than half of health professionals have high levels of burnout concerning the emotional exhaustion subscale. This is consistent with studies conducted in other African and/or Asian nations, such as in Mekelle Ethiopia (55.9%) (30), Iran (55.3%) (31), and Saudi Arabia (54%) (32). However, this level of emotional exhaustion is lower than the findings from the Southern part of Ethiopia (65.2%) (33), Brazil (70.6%) (34), and a pooled prevalence systemic review findings from 45 countries (68.1%) (40). The possible reason for the lower prevalence of burnout in high emotional exhaustion in the current study might be the difference in the cutoff point in high emotional exhaustion (≥ 24 for southern Ethiopia, >14 for Brazil vs. ≥27 in the current study) (33, 34). In contrast, it was higher compared to findings from Addis Ababa (42.0%) (41) and South France (15.8%) (42). The difference in perception of workload might be the plausible reason for the high prevalence of burnout in high EE in the current study as compared to Addis Ababa (high workload; 85.7% in the current study vs. 38.9% in Addis Ababa) (41). Contrarily, the distinction in EE from a study done in France may result from variations in the work environment set-up, patient volume, and working culture. In Ethiopia (43) one medical doctor and nurse are expected to serve populations of 28,847 and 2,299 respectively, whereas the equivalent figure in France is below 325 and 110 (44). Evidence showed that clinicians with high patient volume have a high rate of burnout (33, 45, 46).

In the current study, the magnitude of burnout among health professionals concerning high levels of depersonalization was 38.7%. This scope is comparable with the findings of previous studies done in Addis Ababa, Ethiopia (43.0%) (41), and Saudi Arabia (35%) (32). However, it is lower than the findings from Derdeba, Ethiopia (70.6%) (47) and South Africa (50.0%) (48). The possible reason for the lower prevalence of burnout concerning depersonalization may be derived from the relatively smaller proportion of females (26.0%) that participated in the current study compared to 44.5–55.6% of female participants that were reported in the aforementioned studies. Some evidence showed that depersonalization is more prevalent among females compared to men (49, 50). Another reason for the lower prevalence of depersonalization in the current study may be the presence of a higher number of specialists compared to the above previous studies (47). Conversely, burnout concerning depersonalization was found to be higher compared to previous studies done in Nigeria (15.8%) (51), China (7.5%) (52), and South Khorasan (16.8%) (53). Differences in a study setting might contribute to the differences; the aforementioned studies were conducted among primary healthcare professionals while the current study focused on healthcare workers employed in hospitals, characterized by relatively high workloads (54, 55).

The other dimension of burnout that was assessed in this study is personal accomplishment. According to the findings, 32.2% of the participants have high burnout levels, as displayed by low levels of personal accomplishment. This is consistent with other studies done in Romania (38%) (56), Palestine (34.6%) (57), and a systematic review of findings from low and middle-income countries (31.9%) (58). However, the percentage of high burnout in low personal accomplishment was lower than the findings from Debrebirhan (59.7%) (59), Addis Ababa (45%) (41), and among interns at other Ethiopian teaching hospitals (44.4%) (60). A possible reason for the lower prevalence reported in the current study as compared to the aforementioned studies may be the higher number of physicians who participated in the current study. Research on healthcare professionals showed that undergraduate professionals are more prone to burnout compared to post-graduate health professionals including specialists (41).

In contrast, the prevalence of low levels of personal accomplishment is higher compared to studies done in Mekelle Ethiopia (21.8%) (30), Uganda (18.33%) (61), Belgium (10.4%) (62), Germany (21.5%) (63), and Ecuador (18.2%) (64). The reason may be that compared to other studies, the current sample included a relatively higher prevalence of healthcare personnel that also hold managerial positions in addition to their professional duty (17%) compared to a much lower level in the aforementioned studies (62, 64). Literature findings present that managerial strain is positively associated with low personal performance (i.e., a high level of burnout) (65, 66). Another reason might be that the current study was conducted in high patient-load referral hospitals, which is characterized by the increased workload. A high workload was found to have a positive association with low levels of personal accomplishment (46, 67).

### 4.2. The factors that impact health professionals’ burnout

In this study, midwifery health professionals were found to have a higher level of emotional exhaustion and depersonalization, and a lower level of personal accomplishment (burnout sub-scales) as compared to specialists. Moreover, anesthetic and nursing health professionals have a higher level of emotional exhaustion and depersonalization burnout, respectively, as compared to specialists. A systematic review report from Sub-Saharan Africa and Ghana is in line with the current findings (55, 68). In contrast, a study from Addis Ababa Ethiopia, which concentrated on varied types of professions, did not identify an association with any of the burnout dimensions (41).

The other demographic variable that predicted the level of burnout in the current study is the age of the participants. However, the association was found only concerning the depersonalization burnout sub-scale. According to this study, the level of depersonalization increases by 0.235 every one-year increment in participants’ age. This is in line with other studies (31, 41). Similarly, in a study done in Iran age groups of 30–39, 40–49, and ≥ 50 years had a significant correlation with the increased likelihood of burnout compared to those below 30 years of age groups (69). In contrast, a study conducted among physicians in southern Ethiopia presented that the age of the participants had an inverse relationship with both emotional exhaustion and depersonalization burnout sub-scale scores (33). However, in several other studies, the age of participants was not found to have any statistical association with the three burnout sub-scales (59, 70, 71).

Lower levels of participants’ perceptions of professional recognition were found to be positively associated with emotional exhaustion and low levels of personal accomplishment. In contrast, reducing the perception of high workloads was negatively associated with emotional exhaustion and depersonalization. Similar previous studies support these findings; studies done in Ethiopia and Malawi showed that participants who got recognition/support from hospital managers had a lower level of emotional exhaustion compared to their counterparts (33, 72, 73). The previous findings in Ethiopia in the context of workload showed that an increased workload increases the health personnel’s stress at work (74). Similarly, a study in the United Kingdom (UK) reported a high prevalence of burnout among participants with high work overload (10).

In line with a previous study conducted in Ethiopia (75), the current study revealed that reducing participants’ hope of promotion was positively associated with emotional exhaustion. In contrast to the current study, a study in Malawi presented that participants’ hope of future promotion was not significantly associated with emotional exhaustion (73).

Relationship at the workplace is another work-related variable that determines personal accomplishment. The participants’ level of burnout concerning a low personal accomplishment was increased when the participant’s perception of a relationship at the workplace decreased from very good to very bad relations. This is in line with findings from previous studies (75–77). As was found in the current study, previous findings also revealed that participants who have a fear of contracting an illness during their work have higher emotional exhaustion compared to their counterparts (75).

Among this study’s participants, seniority in work (work experience) had no statistical association with all dimensions of burnout. This was also found in previous studies (78). In contrast, a study done in Malaysia found that health professionals working under 10 years have higher levels of burnout compared to those working above 10 years (79). In divergence, other studies showed a higher prevalence of emotional exhaustion among participants who work above 10 years as compared to health professionals that work less than that (31, 53). Similarly, in a study done in Greek (80) a positive association was found between professional work experience and depersonalization and an inverse relationship with personal accomplishment.

### 4.3. Levels of resilience at work

The mean comparison analysis of this study affirms that the level of mean resilience at work score was higher among medical specialists as compared to nurse professionals. The multiple linear regression findings of this study also revealed that midwifery and nurse professionals have 12.26 and 6.66% lower scores of resilience at work respectively, as compared to specialists. This is in line with previous studies that also found that nurses and midwives present lower levels of resilience at work as compared to medical professionals (81, 82). This difference might be derived from the medical specialists’ having more years of education compared to nurses; an increase in education was found to improve the level of resilience at work (83).

Similar to other studies (82), being divorced and widowed is associated with a lower level of resilience at work, as compared to married health professionals. The multiple linear regression analysis also revealed that participants that are divorced or widowed have a lower mean of resilience at work as compared to their married counterparts.

In addition to the demographic variables, the working environment-related variable (perceived professional recognition) is associated with the resilience mean score. Participants in the current study who have a good perception of professional recognition were found to have a higher level of mean resilience score as compared to those who have a neutral or poor perception of professional recognition. Previous evidence showed that professional recognition and support can increase professionals’ resilience levels in their work (33, 84).

In line with findings from previous studies, resilience at work was found in the current study to have a negative association with emotional exhaustion and depersonalization, and a positive association with personal accomplishment (39, 85–88). Increased professional resilience has an impact on reducing emotional exhaustion, and increasing clinicians’ work engagement, as well as enhancing function when facing challenges at the workplace (89). Health personnel with a high level of ‘resilience at work’ have a negative association with psychological distress and burnout (90). In contrast, experiencing a high level of job-related stress and burnout is positively associated with a high-level health professional turnover and dissatisfaction (91).

### 4.4. The variance explained by the model

The adjusted R-square of the four models ranged from 8.9 to 15.9% (Figures 2-5). This reflects a very small amount of the mean variation of the four outcome variables (emotional exhaustion, depersonalization, personal accomplishment, and Resilience at work) explained by the predictor variables collectively in the model. This small R-square value might be due to different reasons; The first reason might be important predictor variables like; substance use, family size, role in the family, and house ownership not included in the model that might have a higher capacity to explain the mean variation of those outcome variables. The second reason for the low R-square might also be secondary to the difficulty in explaining human behavior. In most cases, a mall R-square value is considered a sign of a bad model, but it is not always true. It depends on the type of the problem being solved; to explain materials high R-square value is recommended, but in some problems such as human behavior, the model with a small R-square value can be considered a good model (92). As a result, the current model could be a good model to predict the outcome variables with the existing low R-square.

### 4.5. Limitations of the study

This study has some limitations; due to the cross-sectional nature; the study does not show the cause-effect relationships between the predictor and the outcome variables. Moreover, due to the small sample size, there is limited generalizability of the current study findings. Since the study targeted specific specialties, the selection bias cannot be excluded. More exploration of working environment-related predictor variables by using a qualitative study design might be useful to identify additional relevant factors.

## 5. Conclusion

This study revealed that most of the health professionals who are working in Ethiopian hospitals experience one or more forms of burnout. Around one-fourth of health professionals, face an overall burnout syndrome. In the current study, a low mean score of ‘resilience at work’ was reported. This may have a negative influence on organizational performance. This study also revealed that professional burnout and resilience at work are inversely correlated. Therefore, efforts should be invested to increase the level of resilience at work and promote professional recognition, as well as reduce high workloads in the strive of reducing burnout. Moreover, all Ethiopian hospitals should learn from this finding, and invest efforts to reduce burnout and enhance resilience at work by strengthening professional recognition, the hope of promotion, relationships at work, and reducing high workload. Further research concerning burnout and resilience at work is recommended by incorporating additional variables such as the presence of comorbidities, family-related challenges, and behavioral variables like substance use. Moreover, intervention-based studies are recommended for assessing the effect of training on the area of resilience and burnout.

## Data availability statement

The raw data supporting the conclusions of this article will be made available by the authors, without undue reservation.

## Ethics statement

The studies involving human participants were reviewed and approved by Ethical Review Board of the Rabin Medical Center and Tel-Aviv University. The patients/participants provided their written informed consent to participate in this study.

## Author contributions

YG and BA were conceived and designed for the study to conduct the analysis. YG, BA, and KH were equally involved in the interpretation and writing of the results. All authors read and approve the final manuscript.

## Conflict of interest

The authors declare that the research was conducted in the absence of any commercial or financial relationships that could be construed as a potential conflict of interest.

## Publisher’s note

All claims expressed in this article are solely those of the authors and do not necessarily represent those of their affiliated organizations, or those of the publisher, the editors and the reviewers. Any product that may be evaluated in this article, or claim that may be made by its manufacturer, is not guaranteed or endorsed by the publisher.

## Abbreviations

EE: Emotional Exhaustion
Gyn/Obs: Gynecologic and Obstetrics
MBI-HSS: Maslach’s burnout inventory human service survey
PA: Personal Accomplishment
TGSH: Tibebe-Ghion Specialized Hospital
UoGSRH: University of Gondar comprehensive Specialized referral Hospital

## References

[1] Portela GZ Fehn AC Ungerer RLS Poz MRD. Human resources for health: global crisis and international cooperation. Cienc Saude Coletiva. (2017) 22:2237–46. doi: 10.1590/1413-81232017227.02702017, PMID: 28724005

[2] Witter S Bertone MP Chirwa Y Namakula J So S Wurie HR. Evolution of policies on human resources for health: opportunities and constraints in four post-conflict and post-crisis settings. Confl Heal. (2016) 10:1–18. doi: 10.1186/s13031-016-0099-0 PMC5241914 28115986

[3] World Health Organization. Global strategy on human resources for health: workforce 2030. Geneva: World Health Organization (2016).

[4] Jackson J Vandall-Walker V Vanderspank-Wright B Wishart P Moore SL. Burnout and resilience in critical care nurses: a grounded theory of managing exposure. Intens Crit Care Nurs. (2018) 48:28–35. doi: 10.1016/j.iccn.2018.07.002, PMID: 30033214

[5] Olson K Kemper KJ Mahan JD. What factors promote resilience and protect against burnout in first-year pediatric and medicine-pediatric residents? J Evid Based Complement Altern Med. (2015) 20:192–8. doi: 10.1177/2156587214568894, PMID: 25694128

[6] Bianchi R Schonfeld IS Laurent E. Is it time to consider the “burnout syndrome” a distinct illness? Front Public Health. (2015) 3:158. doi: 10.3389/fpubh.2015.00158 26106593 PMC4459038

[7] Maslach C Leiter MP. Understanding the burnout experience: recent research and its implications for psychiatry. World Psychiatry. (2016) 15:103–11. doi: 10.1002/wps.20311 27265691 PMC4911781

[8] Martínez-López JÁ Lázaro-Pérez C Gómez-Galán J. Predictors of burnout in social workers: the COVID-19 pandemic as a scenario for analysis. Int J Environ Res Public Health. (2021) 18:5416. doi: 10.3390/ijerph18105416, PMID: 34069394 PMC8158736

[9] Habadi AI Alfaer SS Shilli RH Habadi MI Suliman SM Al-Aslany SJ. The prevalence of burnout syndrome among nursing staff working at king Abdulaziz university hospital, Jeddah, Saudi Arabia, 2017. Divers Equal Health Care. (2018) 15:122. doi: 10.21767/2049-5471.1000165

[10] Imo UO. Burnout and psychiatric morbidity among doctors in the UK: a systematic literature review of prevalence and associated factors. BJPsych Bull. (2017) 41:197–204. doi: 10.1192/pb.bp.116.054247, PMID: 28811913 PMC5537573

[11] Mohammed KA-M Ali EG Youssef IM Fahmy MT Haggag WE. Burnout and personality among Egyptian residents. Arab J Psychiatry. (2013) 44:1–13. doi: 10.12816/0001373

[12] Passi V. Developing resilience throughout the continuum of medical education. Perspect Med Educ. (2014) 3:329–31. doi: 10.1007/S40037-014-0140-1, PMID: 25395227 PMC4235806

[13] Kemp S Hu W Bishop J Forrest K Hudson JN Wilson I. Medical student wellbeing – a consensus statement from Australia and New Zealand. BMC Med Educ. (2019) 19:69. doi: 10.1186/s12909-019-1505-2, PMID: 30832630 PMC6399899

[14] Cawich SO Pooran S Amow B Ali E Mohammed F Mencia M. Impact of a medical university on laparoscopic surgery in a service-oriented public hospital in the Caribbean. Risk Manag Healthc Policy. (2016) 9:253–60. doi: 10.2147/RMHP.S89724, PMID: 27895521 PMC5118042

[15] Montero-Marin J Prado-Abril J Piva Demarzo MM Gascon S García-Campayo J. Coping with stress and types of burnout: explanatory power of different coping strategies. PLoS One. (2014) 9:e89090. doi: 10.1371/journal.pone.0089090, PMID: 24551223 PMC3923838

[16] Cooper AL Brown JA Rees CS Leslie GD. Nurse resilience: a concept analysis. Int J Ment Health Nurs. (2020) 29:553–75. doi: 10.1111/inm.12721 32227411

[17] King DD Newman A Luthans F. Not if, but when we need resilience in the workplace. J Organ Behav. (2016) 37:782–6. doi: 10.1002/job.2063

[18] Southwick SM Bonanno GA Masten AS Panter-Brick C Yehuda R. Resilience definitions, theory, and challenges: interdisciplinary perspectives. Eur J Psychotraumatol. (2014) 5:25338. doi: 10.3402/ejpt.v5.25338, PMID: 25317257 PMC4185134

[19] Robertson HD Elliott AM Burton C Iversen L Murchie P Porteous T. Resilience of primary healthcare professionals: a systematic review. Br J Gen Pract. (2016) 66:e423–33. doi: 10.3399/bjgp16X685261, PMID: 27162208 PMC4871308

[20] Howe A Smajdor A Stöckl A. Towards an understanding of resilience and its relevance to medical training. Med Educ. (2012) 46:349–56. doi: 10.1111/j.1365-2923.2011.04188.x, PMID: 22429170

[21] Caza BB Milton L. Resilience at work: building capacity in the face of adversity In: Cameron K Spreitzer G, editors. The Oxford handbook of positive organizational scholarship. New York: Oxford University Press (2012)

[22] Stoffel JM Cain J. Review of grit and resilience literature within health professions education. Am J Pharm Educ. (2018) 82:6150. doi: 10.5688/ajpe6150, PMID: 29606705 PMC5869747

[23] Foster K Cuzzillo C Furness T. Strengthening mental health nurses' resilience through a workplace resilience programme: a qualitative inquiry. J Psychiatr Ment Health Nurs. (2018) 25:338–48. doi: 10.1111/jpm.12467, PMID: 29920873

[24] Harolds JA Parikh JR Bluth EI Dutton SC Recht MP. Burnout of radiologists: frequency, risk factors, and remedies: a report of the ACR Commission on human resources. J Am Coll Radiol. (2016) 13:411–6. doi: 10.1016/j.jacr.2015.11.003 26768546

[25] Kumar S. Burnout and doctors: prevalence, prevention and intervention. Healthcare. (2016) 4:37. doi: 10.3390/healthcare4030037 27417625 PMC5041038

[26] Al-Hawari MA Bani-Melhem S Quratulain S. Do frontline employees cope effectively with abusive supervision and customer incivility? Testing the effect of employee resilience. J Bus Psychol. (2020) 35:223–40. doi: 10.1007/s10869-019-09621-2

[27] Federal Democratic Republic of Ethiopia Ministry of Health *HSTP Health sector transformation plan 2015/16-2019/20 (2008-2012 EFY)*. Federal Democratic Republic of Ethiopia Ministry of Health (2015).

[28] Maslach C. *Maslach burnout inventory-human services survey (MBI-HSS)*. MBI Manual (1996), pp. 192–198.

[29] Yf G Luo Y Lam L Cross W Plummer V Jp Z. Burnout and its association with resilience in nurses: a cross-sectional study. J Clin Nurs. (2018) 27:441–9. doi: 10.1111/jocn.13952 28677270

[30] Redae GH Dai Y-C. Prevalence and associa ted factors of burnout syndrome among he althcare workers in public and private hospitals in Mek elle City. Ethiopia South Sudan Med J. (2019) 12:17–20.

[31] Zarei E Ahmadi F Sial MS Hwang J Thu PA Usman SM. Prevalence of burnout among primary health care staff and its predictors: a study in Iran. Int J Environ Res Public Health. (2019) 16:2249. doi: 10.3390/ijerph16122249, PMID: 31242691 PMC6616853

[32] Aldrees TM Aleissa S Zamakhshary M Badri M Sadat-Ali M. Physician well-being: prevalence of burnout and associated risk factors in a tertiary hospital, Riyadh. Saudi Arabia Ann Saudi Med. (2013) 33:451–6. doi: 10.5144/0256-4947.2013.451, PMID: 24188938 PMC6074879

[33] Lrago T Asefa F Yitbarek K. Physicians' burnout and factors in southern Ethiopia affecting it. Ethiop J Health Sci. (2018) 28:589–98. doi: 10.4314/ejhs.v28i5.10, PMID: 30607074 PMC6308775

[34] Boni RAS Paiva CE De Oliveira MA Lucchetti G Fregnani JHTG Paiva BSR. Burnout among medical students during the first years of undergraduate school: prevalence and associated factors. PLoS One. (2018) 13:e0191746. doi: 10.1371/journal.pone.0191746, PMID: 29513668 PMC5841647

[35] Pedersen AF Ingeman ML Vedsted P. Empathy, burn-out and the use of gut feeling: a cross-sectional survey of Danish general practitioners. BMJ Open. (2018) 8:e020007. doi: 10.1136/bmjopen-2017-020007, PMID: 29490966 PMC5855338

[36] Hailay A Aberhe W Mebrahtom G Zereabruk K Gebreayezgi G Haile T. Burnout among nurses working in Ethiopia. Behav Neurol. (2020) 2020:1–9. doi: 10.1155/2020/8814557, PMID: 33123299 PMC7586184

[37] Winwood PC Colon R McEwen K. A practical measure of workplace resilience: developing the resilience at work scale. J Occup Environ Med. (2013) 55:1205–12. doi: 10.1097/JOM.0b013e3182a2a60a 24064782

[38] Foureur M Besley K Burton G Yu N Crisp J. Enhancing the resilience of nurses and midwives: pilot of a mindfulnessbased program for increased health, sense of coherence and decreased depression, anxiety and stress. Contemp Nurse. (2013) 45:114–25. doi: 10.5172/conu.2013.45.1.114, PMID: 24099232

[39] Jose S Dhandapani M Cyriac MC. Burnout and resilience among frontline nurses during COVID-19 pandemic: a cross-sectional study in the emergency department of a tertiary care center, North India. Indian J Crit Care Med. (2020) 24:1081–8. doi: 10.5005/jp-journals-10071-23667, PMID: 33384515 PMC7751034

[40] Rotenstein LS Torre M Ramos MA Rosales RC Guille C Sen S. Prevalence of burnout among physicians: a systematic review. JAMA. (2018) 320:1131–50. doi: 10.1001/jama.2018.12777, PMID: 30326495 PMC6233645

[41] Fentie ET Daba Wami S Guyasa KG. Prevalence of burnout syndrome and associated factors among health care workers at public hospitals in Addis Ababa, Ethiopia: results from a cross-sectional study. Int J Ment Health. (2021) 50:368–80. doi: 10.1080/00207411.2021.1946904

[42] Moukarzel A Michelet P Durand A-C Sebbane M Bourgeois S Markarian T. Burnout syndrome among emergency department staff: prevalence and associated factors. Biomed Res Int. (2019) 2019:1–10. doi: 10.1155/2019/6462472, PMID: 30800675 PMC6360614

[43] Alebachew A Waddington C. *Human resources for Health reforms*. Background paper, Ethiopia. (2015).

[44] Chevreul K Brigham B Durand-Zaleski I Hernández-Quevedo C. France: Health system review. Health Syst Transit. (2015) 17:1–218. 26766545

[45] Montgomery A Spânu F Băban A Panagopoulou E. Job demands, burnout, and engagement among nurses: a multi-level analysis of ORCAB data investigating the moderating effect of teamwork. Burn Res. (2015) 2:71–9. doi: 10.1016/j.burn.2015.06.001, PMID: 26877971 PMC4710673

[46] Waddimba AC Nieves MA Scribani M Krupa N Jenkins P May JJ. Predictors of burnout among physicians and advanced-practice clinicians in Central New York. J Hosp Admin. (2015) 4:21–30. doi: 10.5430/jha.v4n6p21

[47] Dechasa DB Worku T Baraki N Merga BT Asfaw H. Burnout and associated factors among nurses working in public hospitals of Harari region and Dire Dawa administration, eastern Ethiopia. A cross sectional study. PLoS One. (2021) 16:e0258224. doi: 10.1371/journal.pone.0258224, PMID: 34714836 PMC8555845

[48] Van der Walt N Scribante J Perrie H. Burnout among anaesthetists in South Africa. South Afr J Anaesth Analg. (2015) 21:169–72. doi: 10.1080/22201181.2015.1102798

[49] Naidoo T Tomita A Paruk S. Burnout, anxiety and depression risk in medical doctors working in KwaZulu-Natal Province, South Africa: evidence from a multi-site study of resource-constrained government hospitals in a generalised HIV epidemic setting. PLoS One. (2020) 15:e0239753. doi: 10.1371/journal.pone.0239753, PMID: 33052921 PMC7556533

[50] Okwaraji F En A. Burnout and psychological distress among nurses in a Nigerian tertiary health institution. Afr Health Sci. (2014) 14:237–45. doi: 10.4314/ahs.v14i1.37, PMID: 26060486 PMC4449076

[51] Lasebikan VO Oyetunde MO. Burnout among nurses in a Nigerian general hospital: prevalence and associated factors. Int Sch Res Not. (2012) 2012:1–6. doi: 10.5402/2012/402157 PMC3350958 22619733

[52] Qiao Z Chen L Chen M Guan X Wang L Jiao Y. Prevalence and factors associated with occupational burnout among HIV/AIDS healthcare workers in China: a cross-sectional study. BMC Public Health. (2016) 16:1–7. doi: 10.1186/s12889-016-2890-7 27079376 PMC4832489

[53] Bijari B Abassi A. Prevalence of burnout syndrome and associated factors among rural health workers (Behvarzes) in South Khorasan. Iran Red Crescent Med J. (2016) 18:e25390. doi: 10.5812/ircmj.25390, PMID: 28180014 PMC5286445

[54] Kotb AA Mohamed KA-E Kamel MH Ismail MAR Abdulmajeed AA. Comparison of burnout pattern between hospital physicians and family physicians working in Suez Canal university hospitals. Pan Afr Med J. (2014) 18:18. doi: 10.11604/pamj.2014.18.164.3355 25422682 PMC4239452

[55] Dubale BW Friedman LE Chemali Z Denninger JW Mehta DH Alem A. Systematic review of burnout among healthcare providers in sub-Saharan Africa. BMC Public Health. (2019) 19:1247. doi: 10.1186/s12889-019-7566-7, PMID: 31510975 PMC6737653

[56] Hagau N Pop RS. Prevalence of burnout in Romanian anaesthesia and intensive care physicians and associated factors. J Rom Anest Terap Int. (2012) 19:117–24.

[57] Hamdan M AaA H. Burnout among workers in emergency Departments in Palestinian hospitals: prevalence and associated factors. BMC Health Serv Res. (2017) 17:407. doi: 10.1186/s12913-017-2356-3 28619081 PMC5472878

[58] Wright T Mughal F Babatunde OO Dikomitis L Mallen CD Helliwell T. Burnout among primary health-care professionals in low-and middle-income countries: systematic review and meta-analysis. Bull World Health Organ. (2022) 100:385–401. doi: 10.2471/BLT.22.288300, PMID: 35694622 PMC9178426

[59] Haile YG Senkute AL Alemu BT Bedane DM Kebede KB. Prevalence and associated factors of burnout among Debre Berhan University medical students: a cross-sectional study. BMC Med Educ. (2019) 19:413. doi: 10.1186/s12909-019-1864-8, PMID: 31703674 PMC6842173

[60] Fisseha H Mulatu HA Kassu RA Yimer SN Woldeyes E. Burnout and stress among interns in an Ethiopian teaching hospital: prevalence and associated factors. Ethiop Med J. (2021) 59:1929

[61] Anita AA Kizito O. Factors associated with Burnout among nurses at international hospital Kampala (IHK). Int J Stud Nurs. (2020) 5:32. doi: 10.20849/ijsn.v5i4.840

[62] Geuens N Braspenning M Van Bogaert P Franck E. Individual vulnerability to burnout in nurses: the role of type D personality within different nursing specialty areas. Burn Res. (2015) 2:80–6. doi: 10.1016/j.burn.2015.05.003

[63] Dreher A Theune M Kersting C Geiser F Weltermann B. Prevalence of burnout among German general practitioners: comparison of physicians working in solo and group practices. PLoS One. (2019) 14:e0211223. doi: 10.1371/journal.pone.0211223, PMID: 30726284 PMC6364915

[64] Ramírez MR Otero P Blanco V Ontaneda MP Díaz O Vázquez FL. Prevalence and correlates of burnout in health professionals in Ecuador. Compr Psychiatry. (2018) 82:73–83. doi: 10.1016/j.comppsych.2017.11.011, PMID: 29444479

[65] Wong CA Spence Laschinger HK. The influence of frontline manager job strain on burnout, commitment and turnover intention: a cross-sectional study. Int J Nurs Stud. (2015) 52:1824–33. doi: 10.1016/j.ijnurstu.2015.09.006, PMID: 26394531

[66] Humphries N Morgan K Conry MC McGowan Y Montgomery A McGee H. Quality of care and health professional burnout: narrative literature review. Int J Health Care Qual Assur. (2014) 27:293–307. doi: 10.1108/IJHCQA-08-2012-0087 25076604

[67] Hämmig O. Explaining burnout and the intention to leave the profession among health professionals–a cross-sectional study in a hospital setting in Switzerland. BMC Health Serv Res. (2018) 18:1–11. doi: 10.1186/s12913-018-3556-1 30340485 PMC6194554

[68] Odonkor ST Frimpong K. Burnout among healthcare professionals in Ghana: a critical assessment. Biomed Res Int. (2020) 2020:1614968. doi: 10.1155/2020/1614968 32280676 PMC7114764

[69] Khodadoost M Zali A Gholamzadeh S Looha MA Akrami F Roodsari SR. Job Burnout and reduced personal accomplishment among Health sector employees during COVID-19 pandemic. Health Scope. (2023) 12:e129841. doi: 10.5812/jhealthscope-129841

[70] Gan Y Jiang H Li L Yang Y Wang C Liu J. Prevalence of burnout and associated factors among general practitioners in Hubei, China: a cross-sectional study. BMC Public Health. (2019) 19:1–9. doi: 10.1186/s12889-019-7755-4 31791282 PMC6889526

[71] Shams T El-Masry R. Job stress and burnout among academic career anaesthesiologists at an Egyptian university hospital. Sultan Qaboos Univ Med J. (2013) 13:287–95. doi: 10.12816/0003236, PMID: 23862036 PMC3706120

[72] Selamu M Hanlon C Medhin G Thornicroft G Fekadu A. Burnout among primary healthcare workers during implementation of integrated mental healthcare in rural Ethiopia: a cohort study. Hum Resour Health. (2019) 17:1–9. doi: 10.1186/s12960-019-0383-3 31319872 PMC6639922

[73] Kim MH Mazenga AC Yu X Simon K Nyasulu P Kazembe PN. Factors associated with burnout amongst healthcare workers providing HIV care in Malawi. PLoS One. (2019) 14:e0222638. doi: 10.1371/journal.pone.0222638, PMID: 31550281 PMC6759146

[74] Birhanu M Gebrekidan B Tesefa G Tareke M. Workload determines workplace stress among health professionals working in felege-hiwot referral hospital, Bahir Dar, Northwest Ethiopia. J Environ Public Health. (2018) 2018:1–8. doi: 10.1155/2018/6286010, PMID: 30598668 PMC6287167

[75] Biksegn A Kenfe T Matiwos S Eshetu G. Burnout status at work among health care professionals in aTertiary hospital. Ethiop J Health Sci. (2016) 26:101–8. doi: 10.4314/ejhs.v26i2.3, PMID: 27222622 PMC4864338

[76] Cao X Chen L Tian L Diao Y. The effect of perceived organisational support on burnout among community health nurses in China: the mediating role of professional self-concept. J Nurs Manag. (2016) 24:E77–86. doi: 10.1111/jonm.12292, PMID: 25728229

[77] She Z Li B Li Q London M Yang B. The double-edged sword of coaching: relationships between managers' coaching and their feelings of personal accomplishment and role overload. Hum Resour Dev Q. (2019) 30:245–66. doi: 10.1002/hrdq.21342

[78] Negi Y Bagga R. Burnout among nursing professionals in tertiary care hospitals of Delhi. J Health Manag. (2015) 17:163–77. doi: 10.1177/0972063415575802

[79] Huda B. Burnout and its associated factors AMONG nurses in a tertiary hospital, Malaysia. Int J Public Health Clin Sci. (2018) 5:215–27. doi: 10.32827/ijphcs.5.6.215

[80] Sofology M Efstratopoulou M Dunn T. Predicting burnout syndrome in Greek mental health professionals. J Soc Serv Res. (2019) 45:142–9. doi: 10.1080/01488376.2018.1480556

[81] Yusefi AR Daneshi S Davarani ER Nikmanesh P Mehralian G Bastani P. Resilience level and its relationship with hypochondriasis in nurses working in COVID-19 reference hospitals. BMC Nurs. (2021) 20:219. doi: 10.1186/s12912-021-00730-z, PMID: 34727947 PMC8561347

[82] Sánchez-Zaballos M Mosteiro-Díaz MP. Resilience among professional Health Workers in Emergency Services. J Emerg Nurs. (2020) 47:925–932.e2. doi: 10.1016/j.jen.2020.07.007 32962846 PMC7502008

[83] Afshari D Nourollahi-darabad M Chinisaz N. Demographic predictors of resilience among nurses during the COVID-19 pandemic. Work. (2021) 68:297–303. doi: 10.3233/WOR-203376, PMID: 33492260

[84] Epstein RM Krasner MS. Physician resilience: what it means, why it matters, and how to promote it. Acad Med. (2013) 88:301–3. doi: 10.1097/ACM.0b013e318280cff0 23442430

[85] Rushton CH Batcheller J Schroeder K Donohue P. Burnout and resilience among nurses practicing in high-intensity settings. Am J Crit Care. (2015) 24:412–20. doi: 10.4037/ajcc2015291, PMID: 26330434

[86] Gerami Nejad N Hosseini M Mousavi Mirzaei S Ghorbani MZ. Association between resilience and professional quality of life among nurses working in intensive care units. Iran J Nurs. (2019) 31:49–60. doi: 10.29252/ijn.31.116.49

[87] West CP Dyrbye LN Sinsky C Trockel M Tutty M Nedelec L. Resilience and burnout among physicians and the general US working population. JAMA Netw Open. (2020) 3:e209385. doi: 10.1001/jamanetworkopen.2020.9385 32614425 PMC7333021

[88] Yusefi AR Faryabi R Bordbar S Daneshi S Nikmanesh P. Job burnout status andand its relationship with resilience level of healthcare workers during Covid-19 pandemic: a case of southern Iran. Iran J Health Sci. (2021) 9:1–11. doi: 10.18502/jhs.v9i3.7305

[89] Yu F Raphael D Mackay L Smith M King A. Personal and work-related factors associated with nurse resilience: a systematic review. Int J Nurs Stud. (2019) 93:129–40. doi: 10.1016/j.ijnurstu.2019.02.014 30925279

[90] Zou G Shen X Tian X Liu C Li G Kong L. Correlates of psychological distress, burnout, and resilience among Chinese female nurses. Ind Health. (2016) 54:389–95. doi: 10.2486/indhealth.2015-0103 27021058 PMC5054279

[91] Selamu M Thornicroft G Fekadu A Hanlon C. Conceptualisation of job-related wellbeing, stress and burnout among healthcare workers in rural Ethiopia: a qualitative study. BMC Health Serv Res. (2017) 17:412. doi: 10.1186/s12913-017-2370-5, PMID: 28629360 PMC5477383

[92] Muralidhar K. *Demystifying R-squared and adjusted R-squared*. (2021).

